# Allegheny County Medical Society

**Published:** 1889-11

**Authors:** W. F. Knox

**Affiliations:** President, in the Chair


### ALLEGHENY COUNTY MEDICAL SOCIETY.

*Special Meeting September 17th, 1889.*

W. F. KNOX, M. D., PRESIDENT, IN THE CHAIR.

### BIRTH OF A DEAD AND OF A LIVING FŒTUS.

DR. GREEN reported the delivery of a living and thriving fœtus of about seven months, after an easy labor, immediately preceded by the extrusion of a dead fœtus of probably three and a half to four months' gestation. There were two placentæ, one a mass of fat without any trace of vessels, the other normal; each also possessed a separate and distinct sac. This was the second case of the kind he had encountered. In the first the living child was born at term, and the dead one at three or four months.

### GRAVITY OF SOME SEEMINGLY SLIGHT EYE DISORDERS.

DR. ALLEN.—I am often impressed with the importance of seemingly slight eye troubles. A man came to me recently, complaining of a slight flickering before one eye. Examination demonstrated about one-third vision, with complete disappearance of vision from about one-half of the field. He had choroiditis, involving the retina in front. Six weeks' treatment perfectly restored vision. A lady came with a little redness of one eye, a trifling redness. It had existed for ten days. She had just one-sixth vision, and there was an adhesion of the retina; it was a genuine case of iritis without pain. By six weeks' treatment her vision was raised to $\frac{6}{6}$. I have knowledge of a similar case in which a physician, unappreciative of the gravity it involved, passed it off as a slight ailment, with the result of destruction of the eye.

NECESSITY OF CAUTION IN PRESCRIBING FOR UNMARRIED PREGNANT WOMEN.

DR. KEARNS.—In March last, a young woman visited me for the purpose of ascertaining whether or not she was pregnant. I examined her and, concluding that pregnaney existed, told her I could do nothing for her. Some time later she called again for certainty in the matter. I examined only her breasts, and pronounced her pregnant. A few days afterward she aborted, fell into the hands of the police, and was removed to the West Penn Hospital. At her hearing I was called as a witness. The charge was that she had taken pennyroyal pills, sold her by a druggist. Both were acquitted. I desire to emphasize the fact that had I, at the time of her second visit to me, but introduced my finger into her vagina, or had I given her medicine of any kind, I would have made myself liable, perhaps to arrest, certainly to newspaper notoriety as accessory to the abortion.

DR. KŒNIG.—Dr. Kearns is over-cautious. I conceive that an examination under such circumstances is proper and safe. The same is true of giving any medicine that may be indicated in the case by any ailment that accompanies the pregnancy. The druggist puts on file and preserves the doctor's prescription; this is his safeguard. The druggist, however, who sells these pills, or any abortive, exposes himself to unpleasant consequences.

DR. W. C. SHAW.—I keep in my office an assortment of fœtuses in alcohol, and explain to my friends the growth, life, etc., instructing them that, no matter how early they may destroy it, this constitutes a destruction of life. This has a powerful moral effect in preventing the induction of abortion.

DR. BATTEN.—Caution is necessary in all such cases. Recently I gave a young girl a solution of sulphate of magnesia, who came complaining of amenorrhœa and constipation. This was cautious, and the remedy a safe one.

DR. BUCHANAN.—The question whether oil of pennyroyal, or pennyroyal in any shape, is a true abortifacient is one open to doubt. Certainly there have been very severe cases of poisoning from the use of oil of pennyroyal by women far advanced in pregnancy in which the pregnancy was not interrupted. Nevertheless, I wish to call attention to the fact that the law of this State provides that, no difference how harmless a substance may be, if it is sold for the purpose of producing abortion, or if it is recommended in the ambiguous language in which the circulars and advertisements of "pennyroyal pills" are worded, "for the use of ladies," the seller and advertiser are incurring the penalty of the law, and the risk of the penitentiary. No difference how harmless the article may be—if he sells chalk for the purpose of producing miscarriage, or words an advertisement in language which would lead unmarried or married women to believe they are getting abortifacient medicine, then he has broken the law and is subject to the penalty of it.

DR. KEARNS.—With regard to over-caution, I would say that you can't be over-cautious in this matter. In view of the class in which these cases occur, it would be wise for you not to have your bottle or box of pills about when the police come in as they may give you a great deal of trouble.

### SYPHILIS COMMUNICATED TO THE MOTHER BY THE FŒTUS.

DR. BATTEN.—A patient had syphilis thirteen years ago, and during eight years has presented no sign of the disease. He married, and two years after the birth of a fine healthy child, presenting no taint, the mother has secondary symptoms. I am positive she was a good honest woman ; and I know the husband has not had syphilis for thirteen years. Was she inoculated through the child, or through the semen of her husband ? I believe it was through the child.

DR. ALLEN.—There is one caution to be observed in making out data in specific cases : You cannot believe a word either party says about the matter. That has been my experience.

DR. GREEN.—There is another point in such cases : Some persons who contract syphilis have it violently for a number of years. They have well-marked symptoms periodically. I think that, at such times, they would be perfectly competent to inoculate a woman.

DR. KEARNS.—I think it is impossible for the wife to escape tertiary symptoms, and the child is apt to become affected through her. I believe, with Dr. Batten, that the mother becomes contaminated through the child.

### REMARKABLE EMACIATION OF A SYPHILITIC.

DR. WARD reported the case of a man, aged 46, who presented complete consolidation of the left lung and syphilis of the brain. His ordinary weight was 225 pounds, and when he presented himself he weighed 118. His temperature was 100°, his pulse 90. He expectorated profusely, and anorexia was complete. He improved under the mixed treatment with iron, and is recovering his health and his weight.

DR. BLUME.—A married man with several ceildren had primary and secondary syphilis. His wife presented no sign of the disease ; her husband had no intercourse with her while he had symptoms. About fourteen months later she aborted ; she had no metritis, and her abortion was due to infection. Another patient, who had syphilis twenty-one years ago, presents brain symptoms periodically, and with treatment promptly recovers.

### FAILURE OF APPLICATIONS OF MERCURY AND TURPENTINE IN DIPHTHERIA.

DR. KŒNIG.—As I reported to this society some time ago the success I had had in the treatment of diphtheria by local applications of corrosive sublimate and turpentine, I desire to report the following fatal case. This case was the third in the family, two recovering. The local treatment applied was one grain of corrosive sublimate in one ounce of spirit of turpentine. The application should have been made every three hours, but owing to the restlessness of the patient, it was omitted in the night. The primary seat of the membrane was the nares, and there was also a spot as large as a quarter upon the roof of the mouth. The membrane was black, hemorrhagic, and the child died, despite everything I could do, on the fourth day from bleeding at the nose.

CHANCROID SIX AND A HALF WEEKS AFTER EXPOSURE.

DR. McMULLEN.—A patient presented himself with a chancroid, which appeared between six and seven weeks after exposure.

DR. STEWART.—An unusual case ; it was probably a chancre ; the time of appearance of the latter may be as late as two months.

DR. W. C. SHAW.— I have seen three cases of chancre of the lip ; one in a policeman, who contracted it by means of a chew of tobacco given him by a friend ; another in a lady whose husband is a syphilitic, and where the virus contaminated a cold blister, and the last in a young lady, by means of kissing an impure mouth.

DR. STEWART.—I will add one case, contracted by means of the tube of a glass-blower.

Dr. WARD reported a case of chancre of the urethra, followed by the usual secondary symptoms.

